# Supplementary material for: Are carbon emissions trading and green financial instruments synergistic? -Comprehensive quantitative research based on content analysis
Source: PLoS One. 2024 Mar 7;19(3):e0298601. doi: 10.1371/journal.pone.0298601 (PMC10919601; doi:10.1371/journal.pone.0298601)
Supplement: S1 Data — (ZIP) [file pone.0298601.s001.zip › Data/地级市Z_F工作报告.zip!/╣π╢1⁄2/├╖╓▌╩╨/└┤╫╘╣1⁄2╓┌║┼í╛┬φ┐╦╩2╛▌═°í┐_2015.docx]

各位代表：

　　我代表梅州市人民政府向大会作报告，请予审议。请政协委员参议，请其他列席人员提出意见。

2014年工作回顾

　　2014年是梅州夯实发展基础、改善发展条件、积蓄发展后劲的重要一年。一年来，市政府在省委、省政府和市委的正确领导下，在市人大和市政协的监督支持下，围绕振兴发展主线，落实粤东西北和原中央苏区两大振兴政策，扭紧“三大抓手”，聚焦产业和项目建设，全市经济社会发展取得新的成绩。全市生产总值、地方公共财政预算收入、固定资产投资和外贸进出口总额分别达到885.83亿元、85.24亿元、407.51亿元和21.82亿美元，分别增长8.5%、22.9%、39.6%和23.8%，增速均超过全省平均水平。

　　一、交通等重要基础设施不断改善

　　全年完成交通基础设施建设投资72.32亿元，增长18.2%。梅大高速大麻至三河段及东延线建成通车，兴华高速公路建设顺利推进，济广高速平兴段、汕湛高速五华段实现无障碍施工，大潮（含大漳支线）、大丰华等4条拟建高速公路前期工作扎实推进。梅汕高铁前期工作基本完成，鹰梅、浦梅铁路和梅州至河源（龙川）、龙岩客专前期工作有序推进。升级改造国道、省道130.96公里和县乡公路378.6公里。完成梅县机场候机楼改造，新增4个通航点、3条航线，客流量增长62.4%。初步确定新机场场址。278宗重点水利项目建设进展顺利，启动2000公里山区中小河流综合治理工程。梅州粤海水务公司正式运营。粤电大埔电厂动工建设，韩江（高陂）水利枢纽工程、梅州（五华）抽水蓄能电站项目取得实质性进展。全面完成年度投资10.28亿元的电网项目建设。

　　二、产业园区进一步扩容增效

　　理顺广梅产业园管理体制机制，优化园区发展规划，拓展园区空间布局，启动丰华兴梅产业集聚带规划建设，促进市县联动发展。成立广梅投资有限公司，推动园区实体化运营。广梅产业园开发面积扩展到12.89平方公里，园区14个基础设施项目加快推进，新落户工业项目17个、计划总投资31.5亿元。推进各县（市）工业园或产业集聚地的规划建设。全市工业园区实现产值324.69亿元、增加值71.99亿元，分别增长32.1%和34.1%；省级园区规模以上工业增加值26.81亿元，增长22%。深入实施乡贤回乡投资兴业工程，促进产业集聚发展。全市引进项目247个，其中落地项目201个，实际投入资金153.19亿元。

　　三、实体经济发展有新成效

　　工业对经济的拉动作用增强。制定扶持实体经济、县域经济发展的政策，深入实施产业振兴三年行动计划，大力开展引智强企活动，传统优势产业转型升级的步伐加快。全市规模以上工业总产值646.22亿元、规模以上工业增加值206.93亿元，分别增长14.5%和10.7%。烟草、电力、建材、电子信息、机电制造、矿冶加工等六大产业工业增加值174.04亿元，增长10.5%；完成工业技改投资51.91亿元，增长58.7%；新增规模以上工业企业31家、年外贸进出口总额超千万美元企业19家。加强产学研合作，推动企业自主创新，新增4个省级工程技术研发中心，建立梅州工业设计中心。博敏获中国驰名商标。全市专利授权量1609件，梅县区被确定为国家知识产权强县工程试点区。

　　现代服务业发展步伐加快。消费新业态保持良好发展势头，全市社会消费品零售总额499.97亿元，增长11.1%。建成4家电子商务产业园，入园企业160多家，电子商务交易额达115.4亿元，增长55.8%；积极参与“广货网上行”，支持中小企业“上网触电”，500多家中小微企业加入了“百度翔计划”。综合信息基础网络不断完善。休闲旅游、养生保健、文化创意等产业融合发展，新增泰安楼客家文化旅游产业园和百侯名镇旅游区2个国家4A级旅游景区，全市接待旅游总人数2521.7万人次、旅游总收入254.39亿元，分别增长23.8%、26.9%。梅州获“中国最美生态休闲旅游城市”，梅县雁洋成为全国第二个“国际慢城”，丰顺获“中国温泉之城”称号，蕉岭获评“世界长寿乡”，梅县、丰顺获评“中国长寿之乡”。金融对地方经济扶持力度加大，全市各项贷款余额635.46亿元，比年初增长16.1%；存贷比达45.02%，比上年提高0.32个百分点；中小微企业贷款余额增长11%。新增2家村镇银行。风华环保在“新三板”挂牌，9家企业在广州股权交易中心挂牌。

　　农业效益有新提高。完成高标准基本农田建设23.31万亩。粮食生产保持稳定。农业总产值285.87亿元、农业增加值179.47亿元，分别增长4%和4.3%。新一轮扶贫开发“双到”工作成效显著，贫困村村均集体经济收入达5.4万元、贫困户人均纯收入6623元。推动农业产业化发展，重视特色产业基地建设，新增金柚、慈橙、茶叶4.5万亩，新增2家国家级林业龙头企业、34家省级农业（林业）龙头企业和749家农民专业合作社，新登记注册家庭农场236家。农业组织化、信息化、市场化水平不断提高，组建了12个覆盖全市的农业行业协会；完善了农业综合信息服务平台，惠及63.7万用户；动工建设海吉星农产品物流园，搭建客天下农电商产业园，拓宽农副产品销售流通渠道。特色农产品品牌进一步打响，丰顺马图绿茶获评国家地理标志保护产品，开发富硒茶等长寿食品70多种，新增14个省名牌农产品，4个农产品被评为广东“名米、名茶、名果”。

　　四、中心城区扩容提质步伐加快

　　设立嘉应新区管委会，集中力量推动江南新城开发建设。优化江南新城规划。剑英湖扩建工程基本完成，市政路网和安置区加快建设。客都汇商业文化广场建成开业，万达广场、富港东汇城、奥园等一批城市综合体建设进展顺利。芹洋半岛和梅县新城、兴宁新城、丰顺新区等建设扎实推进。做好土地征收储备工作，积极引导金融资本、社会资本参与城镇化建设，成功争取粤东西北振兴发展股权基金和国家开发银行、国家农业发展银行政策性中长期贷款。统筹推进美丽乡村建设，镇村规划编制基本实现全覆盖，完成了“四镇六村”历史文化名镇名村保护规划，打造了一批新农村建设示范片区，建成国家级美丽乡村示范点4个，13个村被评为中国传统村落，数量居全省首位。

　　五、生态保护和社会建设力度加大

　　绿色生态巩固提升。全面推进国家生态文明先行示范区建设。绿化宜林荒山，加强森林资源管护，森林覆盖率达73.55%。成立省首个林业产权流转服务平台，8000亩碳汇造林项目（CCER）获国家核准备案。大力推进大气污染防治，城区环境空气质量保持优良。完成各项节能减排年度计划任务，预计单位生产总值能耗下降3.66%。扎实推进南粤水更清行动，水环境质量达标率保持100%。深入实施城乡环境综合整治工程，全市城镇生活垃圾无害化处理率达85%，创建127个省卫生村，城乡环境得到进一步改善，我市成为全省唯一未报告登革热病例的地级市。国家卫生城市、国家园林城市复审和国家环保模范城市创建工作扎实推进。

　　社会治理成效显著。深入创建平安梅州。社会治安防控体系建设不断加强，“客都民意警务”扎实推进，“六大打击”行动成效突出，刑事、治安案件发案数明显下降。我市群众安全感和政法工作满意度居全省第一，连续7年获评“全省社会管理综合治理优秀市”。社会工作示范镇、示范村、示范社区创建活动和省级社会创新实验基地建设扎实推进，蕉岭县镇级“一办一中心”改革被评为“全省基层社会治理创新优秀项目”。加强社会组织自律自治，新增社会组织321家，注册志愿者增至15.3万人。拓宽与群众沟通的平台，55位市、县（市、区）政府领导和部门负责人上线参加“梅州发布”微访谈、“行风热线”活动，访问量达2540多万人次，“12345”政府服务热线办结率100%，推动解决了一批群众关注的热点难点问题。“梅州发布”获评人民网2014年度“十大办实事政务微博”和广东十大区域特色微信。完善网上信访大厅建设，实现网上信访事项办理可查询、跟踪、监督和评价，开通免费信访绿色邮政，方便群众反映诉求。加强应急演练培训，编制和修订各类应急预案156个。强化安全生产红线意识，完善事故隐患排查治理机制，较大事故起数同比下降25%。

　　其他各项事业协调发展。依托广州帮扶，新一轮对口帮扶工作成效明显，动工建设产业项目79个，财政帮扶资金9.53亿元。坚持教育优先发展，教育强县、强镇实现全覆盖，梅江区、梅县区、平远县和蕉岭县获认定“全国义务教育发展基本均衡县”。国家级示范性高中带动作用增强，薄弱高中改造提升工作稳步推进。积极创建国家级客家文化（梅州）生态保护区，新增省级非遗项目传承人12名。开辟了群众喜闻乐见的汉剧、山歌演艺平台。山歌剧《客魂·家风》、汉剧《诗娘》分别获省艺术节优秀剧目一等奖、特别奖。第二届客家文化创意产品博览会成功举办，影响力进一步扩大。完成振兴足球之乡十年规划第一阶段任务。强化责任考核，稳定适度低生育水平。国防教育、兵役动员、双拥等工作扎实推进，外事侨务、港澳和对台工作有新进步，民族宗教、人事编制、妇女儿童和监察、审计、统计、物价、法制、供销、气象、残联、打私、人防、档案、方志等工作均取得新成绩。

　　办成了一批民生实事。坚持民生为重，财政支出向民生倾斜，民生领域支出占公共财政预算支出的73.3%，增长33.2%。全力办好十件44项民生实事：提高了底线民生保障项目标准和职工养老保险的企业退休养老金、城乡居民医保年补助、城镇职工医疗保险年最高支付限额、城乡居民医疗保险住院报销比例等社会保障项目标准，职工医疗保险、城乡居民医疗保险与广州6家医院实现联网即时结算，对民办养老机构（含公办民营）给予运营补贴。义务教育标准化学校覆盖率99.1%，动工建设了8所学校或幼儿园，城乡免费义务教育生均公用经费补助标准提高。各县（市）120公共卫生应急指挥中心建设全面完成，为每个乡镇卫生院配置“五个一”医疗设备；提高了人均基本公共卫生服务经费标准，实施偏远乡镇卫生院在编人员岗位津贴补贴政策；地中海贫血筛查率得到提高，重型地中海贫血发生数下降51.7%。完成了各县（市、区）污水处理厂二期工程、梅州城区8米以下道路（巷道）路灯照明（一期工程）以及市级能源管理中心、重过载配电变压器和低电压台区的改造建设；城市建成区数字化城市管理稳步推进。13227户农村低收入住房困难户住房改造任务全面完成，9068套（户）保障性住房、棚户区改造住房开工建设。为66902户城乡低保户和农村五保户提供每月每户15度免费用电，为3000名视力、听力和肢体残疾人免费提供了康复服务，为视障人员免费配发读屏软件。完成了市第三人民医院规划，启动了资产置换和建设工作；为10507名重性精神疾病患者免费发放药物，免费收治了528名贫困重症精神疾病患者。客都大桥动工建设，完成新农村公路路面硬底化改造工程508公里；205国道城区段改造全面完成，渡江津桥修复工程顺利推进，完成了一批交通环岛改造和城区市政道路建设，城北区域的交通拥堵状况得到一定的改善。梅州城区增加72辆新能源公交车，梅县区新增100辆出租车，公共交通的覆盖面进一步扩大。梅江群众文化长廊和一批公园绿地歌舞台建设有序推进，建成30个农村文化俱乐部试点，县级多厅数字影院实现全覆盖，完成12个乡镇（街道）公共电子阅览室、349个行政村（社区）文化室设备和10个社区体育小公园、16个乡镇农民健身工程建设，基本完成12个标准足球场和其他体育设施建设。完成50.85万亩森林碳汇重点生态工程、213公里生态景观林带和24个“乡村绿化美化工程”市级示范点建设，完成21个城区周边森林公园、湿地公园的规划工作。

　　六、政务环境优化提升

　　作风建设得到加强。深入开展党的群众路线教育实践活动，严格执行中央八项规定。全市性会议减少36.7%，政府文件减少29.7%；各类领导小组和议事协调机构进一步压缩。狠刹公款送礼、公款吃喝、奢侈浪费，全市“三公”经费支出同比降低28.4%。节庆、庆典、论坛、展会等活动大幅度减少，大力整治超标配备公车、多占办公用房、新建滥建楼堂馆所等问题，严肃查处了54名违反“八项规定”和存在“四风”突出问题的干部，对失职渎职、工作落实不力的66名责任人和36个责任单位进行了责任追究。进一步完善市、县、镇、村四级政务服务体系，建立健全村级便民服务站1707个，方便了群众办事。

　　依法行政工作有新进步。重视法治政府建设，健全重大决策社会稳定风险评估机制，完善重大行政决策程序规定和重大事项集体决策、专家咨询、听证、公示、新闻发布等制度。积极开展依法行政考评工作，“六五”普法工作扎实推进。自觉接受市人大常委会和市政协的监督，2件省人大代表建议、2件省政协委员提案和64件市人大代表建议、243件市政协委员提案全部办复。认真听取各民主党派、工商联、无党派人士及工青妇等人民团体意见。全市114个镇（街）、1443个村（居）委设立了村级法律援助工作站。

　　社会和市场活力增强。积极稳妥推进改革，加大简政放权力度，清理压减行政审批事项53项，向社会转移职能事项40项。市政府部门积极落实155项“微改革、微创新”项目，行政效率明显提高。商事制度改革全面铺开，企业登记注册整体时限提速75%；新登记各类市场主体2.7万户，其中注册资本千万元以上企业414户，分别增长31.2%和156%。鼓励和引导民间投资健康发展，民间投资招标重大项目有新突破。改进预算管理制度，增加预算编报范围。制定政府投资管理办法，完善项目管理。成立市公共资源交易中心，强化政府采购、工程建设、产权交易和土地出让等重点领域监管。深化农村金融改革创新，完成农村土地确权登记颁证蕉岭县试点工作，成立全省首家县级农村产权交易中心。公立医院综合改革试点实现县（市）全覆盖，中山大学粤东医院正式运营。

　　各位代表！过去一年的成绩，凝聚着全市人民的智慧和汗水，饱含着省委、省政府的关怀，离不开市委的正确领导和市人大、市政协的监督支持，也得益于广州市的鼎力帮扶。在此，我谨代表市政府，向全市人民、中央和省属驻梅单位、驻梅部队，向所有关心支持梅州发展的海外侨胞、港澳台同胞和社会各界朋友，表示衷心的感谢并致以崇高的敬意！

　　回顾过去一年的工作，我们也清醒地认识到，我市经济社会发展离广大人民群众期盼还有不小的差距。尤其是经济增长速度未能实现预期目标，客观上是受经济大环境的影响，二三产业增速减缓，特别是工业增长未达预期，拉低了经济增长速度，其中烟草、水泥等骨干传统企业受产业政策的限制，对经济增长的贡献有所减弱；主观原因是市政府对新常态下经济发展的新特点、新变化和新趋势认识不足，对经济发展面临的困难和问题估计不足，各级政府克服困难解决问题的决心不足、办法不多。加上全市工业技改投资长期处于低位，企业推动技术改造和转型升级的积极性、主动性不强，导致企业的经济效益和抗风险能力较差，经济持续增长缺乏有力支撑。制约我们全市经济社会发展的突出问题还有，出省出海的快速通道尚未全面打通，与周边地区的经济互补性和产业关联度低；全市城镇化水平明显低于全省平均水平，城区和各县城产业和人口的承载能力弱，聚积经济发展要素的能力差；县域经济整体上比较落后；一些政府工作人员担当精神、责任意识和服务水平有所欠缺，极少数公职人员不作为或乱作为，等等。我们将正视这些困难和问题，切实采取有力措施加以解决。

2015年工作安排

　　各位代表！2015年，是全面深化改革、加快梅州振兴发展的关键之年，也是推动依法治市的开局之年、“十二五”的收官之年。当前，我市既面临着加快振兴发展、改善民生的重任，又肩负着守护好绿水青山的使命。我们既要看到宏观经济形势仍有许多不稳定、不确定的因素，保持经济持续较快发展的难度加大，但也应充分认识到，稳中求进仍然是我国经济发展的总基调，宏观政策的连续性和稳定性继续保持，经济发展长期向好的基本面没有改变。对梅州而言，发展面临着诸多有利条件，《赣闽粤原中央苏区振兴发展规划》和省委、省政府促进粤东西北振兴发展的一系列扶持政策，将进一步实施到位；广州对口帮扶力度加大，更多的成长性项目逐步落地，经济增长动力增强。只要我们乘势而上、顺势而为，抓住关键、夯实基础，就一定能够在新常态下实现新作为，推动梅州加快振兴发展。

　　今年政府工作的总体要求：深入贯彻落实党的十八届三中、四中全会、中央经济工作会议、习近平总书记系列重要讲话和省委、市委全会精神，以胡春华书记、朱小丹省长来梅调研讲话精神为指导，顺应经济发展新常态，围绕振兴发展主线，落实粤东西北和原中央苏区两大振兴政策，紧紧扭住“三大抓手”，以提升经济质量和效益为中心，突出发展实体经济，促进产业集聚，推动民生改善，守住绿水青山，实现绿色发展和惠民发展，努力建设富庶美丽和谐幸福梅州。

　　今年全市经济社会发展的主要预期目标是：生产总值增长10%左右；公共财政预算收入增长15%以上；固定资产投资增长30%以上；单位生产总值能耗下降2.35%；人口自然增长率控制在7.5‰以内；居民收入增长与经济增长基本同步。

　　实现今年的目标任务，重点是抓好六方面工作：

　　一、致力发展壮大实体经济

　　要把振兴实体经济、吸引产业集聚，作为发展梅州经济的首要任务抓紧抓好。

　　推进丰华兴梅产业集聚带建设。充分用好扶持苏区发展政策，推动产业集聚带建设上升为省级战略。高起点编制规划，统筹推进多规合一。突出基础先行，动工建设梅城至畲江快速干线，加快推进产业带路网建设，促进产业带园区之间互联互通。发挥广梅产业园的核心带动作用，积极支持配合广州市工作团队，加快推进保税、仓储、物流园区和中心商务区建设，完善园区及周边城镇基础设施和公共服务配套建设，提升园区承载力和产业集聚能力。力争全市产业园区规模以上工业总产值、工业增加值，均增长30%以上。

　　培育发展主导产业。发挥电力、建材、烟草和电子信息等产业基础优势，做大做强骨干企业，打造支柱产业，促进产业集聚发展。着力抓好粤电大埔电厂和梅州（五华）抽水蓄能电站等一批电力项目，加快培育百亿电力产业。围绕建成千万吨水泥生产基地，推进龙头企业技改项目建设，打造百亿建材产业集群。扎实推进梅州卷烟厂技改项目，推动烟草产业由规模效益向结构效益转型发展。围绕打造中国电声之都，大力推进电声创业园、省质量监督电声产品检验站等项目建设，推动电声产业集群、集约发展。以本地骨干企业为依托，吸引上下游配套企业聚集，打造电子信息和装备制造业集群。

　　推动现代服务业提档升级。坚持扩内需稳外需，加快推进现代服务业升级发展、集聚发展。发挥文化和生态等特色优势，推进客家文化旅游特色区建设，提升文化旅游产业的质量和效益。加快雁洋生态旅游园等重点生态景区开发建设，力争建成一批具有特色的生态型度假区；完善旅游配套设施，延长旅游产业链条，增强旅游业综合效益。致力推动文化创意和设计服务与产业融合发展，提升文化软实力和产业竞争力。大力发展电子商务，重点推进农产品电子商务产业园、电商示范基地等平台建设，培育引进一批引领带动能力强的电商企业。推动电子商务和快递业联动发展，鼓励本地企业创建第三方电子商务平台，开展跨境电子商务，开拓国内外市场。深入实施万村千乡市场工程，加快推进物流园区、物流通道建设，打造一批区域性物流中心和商品交易中心。大力推动云计算IDC建设，着力打造“数字梅州”，建设“智慧城市”。鼓励电声、陶瓷、家具、工艺、汽车配件等外向型企业抱团发展，开拓国外新兴市场，提高市场份额。

　　搭建服务企业要素平台。完成国家水泥及制品监督检验检测中心、省质量监督稀土产品检验站建设。积极构建电声、电子信息、五金制品等特色产业综合公共服务平台。加强政银企合作，落实小微企业税收等优惠政策，设立中小微企业信贷风险补偿基金，建立全市统一的企业信用体系，破解中小微企业融资难问题。强化金融服务和创新，鼓励证券机构参与企业股改，支持企业上市和在“新三板”或区域股权交易中心挂牌。落实保险“新国十条”，提高企业防范和化解风险能力。深入开展质量强市工作，鼓励企业创建商标品牌，提高产品附加值。继续实施民营企业家素质提升工程。

　　突出抓好招商引资。以举办第四届世界客商大会为契机，深入实施乡贤回乡投资兴业工程，聚焦行业领军企业，综合实施领导招商、乡情招商、以商引商、产业链招商和委托专业招商等措施，着力引进一批带动能力强、发展前景好的大项目，实现“招一个、引一串、带一片”。坚持对外招商和促进现有企业增资扩产并重，加强对现有企业的跟踪服务；支持企业用好省扶持企业技术改造政策，实施技改强企工程，支持百家工业企业技术改造。

　　推动区域经济合作。依托梅州独特的区位优势，加强与珠三角、汕潮揭、厦漳泉和其他周边城市的沟通对接，建立健全人才、市场、资源等要素双向聚合的长效联动机制，推动交通、产业、文化旅游、生态建设等方面的合作。借助深圳、广州和汕头深水港口，依托广梅产业园规划建设的物流园和保税服务区，探索合作共建“无水港”，打通铁海联运通道，努力降低园区物流成本。扎实推进丰顺韩江生态文化经济带规划建设。积极抢抓国家实施“一带一路”发展战略的机遇，发挥我市重点侨乡优势，加强与海外华人华侨的联系，充分利用在外侨胞的资源，拓宽与海外地区的合作空间。

　　二、加快推进以交通为重点的基础设施建设

　　以构建潮汕平原北上拓展腹地的交通枢纽为目标，继续打好交通基础设施建设大会战。

　　全力推进高速公路建设。加快推进济广高速平兴段、汕湛高速五华段建设，确保年底前建成，实现县县通高速目标。全面完成兴华高速公路征地拆迁工作，实现全线动工建设。着力推进梅平、东环、大潮（含大漳支线）、大丰华丰顺至五华段等4条高速公路前期工作，力争年内动工建设。

　　加强铁路、机场的规划建设。大力推进梅汕高铁动工的前期准备工作。推进鹰梅铁路立项，争取纳入中铁总公司今年开工项目。推动梅河高铁规划建设，力争与赣深客专同步立项建设。开展梅州至龙岩高铁前期工作。完成梅县新机场选址，力争早日动工。加强梅县机场口岸建设，创造条件开辟新航线，发挥航空港的综合效应，推动对内对外更宽领域开放。

　　完善市域交通网络。推进4条省道升国道、6条县道升省道的规划建设，提升干线公路等级。力争完成5条县乡公路改造。完成25个国省道“迎国检”项目建设任务。加快畲江客运中心站、兴宁综合物流园建设，确保年内建成运营。抓好韩江三河坝至汕头航道整治，提高航道等级和改善通航条件。

　　谋划推进一批基础设施重大项目。发挥投资拉动作用，谋划储备一批重大产业和基础设施项目，争取列入国家和省的“十三五”规划。推进水利、电力等重大基础设施建设，年内完成510公里中小河流综合治理。动工建设韩江（高陂）水利枢纽工程，规划建设韩江（葛布）水利枢纽工程。粤电大埔电厂首期两台机组年底建成投产。争取梅州（五华）抽水蓄能电站上半年获省核准开工建设。继续抓好农村电网改造。

　　三、务实推进新型城镇化

　　新型城镇化是以城乡统筹、城乡一体、产城互动、节约集约、生态宜居、和谐发展为基本特征的城镇化。它既是经济增长的重要动力，也是改善群众生产生活条件的重要途径。

　　推进中心城区建设。坚持规划引领，重视城市规划的相互衔接，统筹江南新城、芹洋半岛、梅县新城规划，推进“三规合一”。2月底前完成江南新城规划优化工作，年底前完成嘉应新区和中心城区控制性规划，确保“一张图”规划、“一盘棋”建设。全力推进江南新城起步区建设，基本完成6.66平方公里征地拆迁工作，抓好新区路网建设；加快推进安置区建设，年底前实现首批拆迁户回迁；突出产城联动，推动相关产业园区与江南新城互补互动发展，促进人口和产业集聚；根据市场需求，规划建设总部经济聚集区，吸引总部经济相关机构进驻。推进芹洋半岛、梅县新城征地拆迁、安置区和一批基础项目建设。有计划、有步骤改造提升江北老城区，完善优化江南建成区，开展地下管廊改造试点工作，改造东教场体育场和游泳池，不断完善城市公共服务配套功能。充分利用棚户区改造等政策性金融信贷和PPP等融资模式，引导更多民间资金和社会资本投入新型城镇化建设。遵循城市开发建设和经营的规律，控制好新城开发和旧城改造的成本，推动城市建设和经营转入良性轨道。

　　提升县城和中心镇集聚能力。开展县域全域规划编制试点工作，加强对产业和公共服务资源布局的引导，拓展县城发展空间。加快推进兴宁新城和各县县城开发建设，努力提升县城的综合服务能力。支持各县（市、区）推进中心镇的规划建设，加大镇区市政道路、供水供电、污水及垃圾处理等基础设施的投入，增强教育、医疗、文化和社区服务等公共服务的供给，促进县城和中心镇合理分工、功能互补、协同发展。因地制宜发展特色产业，建立完善专业镇服务中小微企业的平台，培育一批特色工业强镇、商业物流镇和文化旅游特色镇。积极推行户籍制度改革，引导农业人口就近城镇化。

　　改善城乡环境。加强新时期爱国卫生工作，巩固国家卫生城市创建成果，综合整治城市“六乱”等环境卫生问题，确保成功通过全国卫生城市复审。实施环境基础设施建设工程，扎实创建国家环保模范城市。综合治理大气污染，深入开展汽车尾气、酒店油烟和建筑工地扬尘等专项整治，完成黄标车、老旧车和高污染燃料锅炉年度淘汰任务，力保市区全年空气质量优良率达91%以上。用好国家节能减排财政政策综合示范城市专项资金，推动工业、建筑和交通运输节能，全面完成节能减排目标任务。加强城区活禽市场管理整治，开展病死畜禽无害化处理工作，争取城镇生活垃圾无害化处理率达90%以上。扎实推进美丽乡村建设，进一步提高村庄规划编制质量，规范农村建房，引导农村住宅建设向中心镇村集聚。完成传统古村落保护规划编制，争取申报一批中国传统村落。大力推进卫生镇村和幸福村居创建活动，农村生活垃圾无害化处理率达到60%；继续实施农村人居环境改善工程，抓好农村环境连片综合整治试点工作，美化净化乡村。

　　四、推动县域经济加快发展

　　县域经济是以县级行政区域为地理空间，以县级行政权为调控主体，以市场为导向，优化资源配置，具有地域特色和功能完备的区域经济。在幅员较大的梅州，县域经济发展的程度，直接影响全市经济的规模与质量。

　　进一步简政放权。落实县域经济扶持办法，编制完成市县（市、区）政府部门第一批权责清单目录。积极向上争取完善县级基本财力保障机制，促进县级财力与事权基本相匹配。扩大县（市、区）经济管理权限，尽可能下放投资项目审批、存量建设用地预审、行政执法检查等权限，最大限度释放县（市、区）抓发展的能量和活力。

　　培育县域特色工业产业。支持各县（市、区）建设1个工业园区或产业集聚地，争取享受省级产业转移园政策优惠。支持各县（市、区）因地制宜培育发展1-2个特色主导产业，引导生产要素向优势产业和龙头企业集聚，重点打造电子信息、机械装备、新型建材、五金机电、食品药品、稀土深加工、特色陶瓷和家具制造等一批县域特色品牌。支持民间资本、社会资本更多地参与县域经济建设，争取设立1家民营银行和新增1家村镇银行，推动2家以上农信社改制为农商行，依法设立一批助力县域经济发展的小额贷款公司。

　　发展精细高效农业。坚持精耕细作，提高土地产出效益。重视粮食生产，不断提高粮食安全保障水平。围绕“一县多品、一镇一品”，大力发展金柚、慈橙、茶叶、高山油茶、优质稻米等绿色产品。因地制宜发展林下经济，规划建设一批林下珍贵药材示范基地和集中连片示范点。打响长寿品牌，做精做优区域特色板块。加快台湾农民创业园、海吉星农产品物流园和现代农业示范区建设。抓好大宗农副产品的深加工，培育和引进一批有品牌、有实力的龙头企业。完善农产品直供直销等平台，健全线上、线下农产品流通网络。鼓励发展家庭农场、专业庄园等新型农业经营主体，新组建一批农民专业合作社。完善疫情预警预报机制，强化农产品质量和食品安全监管。

　　推动农村综合改革。推广蕉岭“三位一体”农村综合改革经验，全面铺开农村土地承包经营权确权登记颁证工作，健全土地经营权流转市场，探索建立“土地银行”、农业产业投资基金，盘活农村土地资源、金融要素，全面建成县镇村农村“三资”管理服务平台。发挥农业协会作用，提高农业社会化服务水平。重视农村基础设施建设，认真实施好“一事一议”项目，积极推进村村通自来水工程建设，加强水库、山塘、农村饮水安全等运行管理，改善农民生产生活条件。深入推进农村精神文明建设，倡导移风易俗，培养良好道德风尚，提高农民综合素质。

　　五、发展社会事业和全力保障民生

　　实现发展惠民，推进社会保障和教育卫生等社会事业均衡发展，是各级政府的重要职责。

　　提升生态环境。坚持绿色发展，加快推进生态文明先行示范区建设。深入开展新一轮“绿满梅州”大行动，加快森林资源培育，优化林分结构，加强生态修复，完成50.85万亩碳汇林建设，确保完成“一消灭三改造”任务。启动林业生态红线划定工作，建立林业生态红线地理空间数据库。依托林业产权流转服务平台，积极推进森林碳汇开发，建立碳汇交易机制，探索排污权、碳排放权有偿使用制度。落实森林防火责任制，加强森林消防专业队伍建设。深入开展南粤水更清行动，推动实施韩江上游跨省界流域污染治理等重大环保工程，确保饮用水源安全，城市污水处理率提高到85%。实行最严格的保护生态环境执法监督制度，提高全社会环境保护和造林绿化美化的责任意识、参与意识和法律意识。

　　推进社会治理创新。深化社会体制改革，扎实推进全市基层社会治理服务平台建设。推进基层自治，发挥村民理事会作用，促进农村稳定和谐。深入宣传和引导公民践行社会主义核心价值观。培育发展一批骨干型社会组织，提高专业社工、志愿者队伍服务水平，推动社会协同共治。巩固提升“平安梅州”成果，深入开展“客都民意警务”，扎实推进群防群治服务队建设，构建立体式的社会治安防控体系。加大力度整治城区交通秩序。推进食品安全示范学校食堂建设，加强对全市供餐人数百人以上学校食堂的动态监管。巩固打假治劣成果。拓宽网上信访平台，积极推进行政复议工作，进一步畅通群众表达诉求的渠道。依法加强安全监管。整合各类应急资源，完善应急处置机制，全面提升综合应急能力。

　　努力提高社会保障水平。积极落实促进就业创业政策，突出抓好大学生等重点群体的就业创业服务，实现新增城镇就业3万人、创业带动就业1万人，组织开展劳动力技能晋升培训3万人次，把城镇登记失业率控制在3.5%以内。进一步抓好社会保险扩面征缴工作，年内职工社保“五大险种”综合参保（含企业离退休）人数达222万人次。推进城乡社会保障体系统筹并轨，健全低保标准动态调整机制和失地农民社会保障制度。积极构建和谐劳动关系，及时、有效预防和化解劳动人事争议。推广居家养老模式，鼓励社会资本参与发展养老事业。

　　促进各项事业协调发展。推动城区优质教育资源向农村学校辐射，实行优质学校与薄弱学校结对帮扶，进一步优化城乡教育资源配置，实现“全国义务教育发展基本均衡县”全覆盖。推进标准化特殊教育学校创建工作。支持嘉应学院加强国际交流与合作，争取办出特色、扩大影响。促进全民健身运动的深入开展，因地制宜建设一批社区体育设施。依托梅州的资源禀赋，促进体育产业发展，举全市之力支持足球事业发展，擦亮足球之乡品牌。深入推进客家文化（梅州）生态保护区建设，办好客家文博会，打造客属地区文创产品展示交易平台。规划新建江南片区三甲医院。动工建设市人民医院负压病房，提升突发传染病应急救治能力。推进县级公立医院改革，在蕉岭、平远开展县域医疗联合体试点工作，努力找到提升县镇医疗卫生公共服务水平的有效路径。加强计划生育工作，确保完成全年人口控制目标任务。重视做好老龄和残疾人事业。完成妇女、儿童发展规划中期阶段任务。加强梅港青少年交流。推动国防动员、国家安全、人防、双拥、对台、气象、防灾减灾、民族宗教和人事编制，以及统计、打私、方志、档案等各项工作取得新成效。

　　完成扶贫开发“双到”任务。借力广州对口帮扶，突出抓好产业扶贫、学技扶贫等造血式扶贫，注重创新体制机制，整合各方资源，增强贫困村、贫困户自身发展能力。年内培训贫困户劳动力4.7万人次以上，确保完成386个重点帮扶村、3.7万贫困户14.6万贫困人口稳定脱贫任务。

　　六、努力建设守法诚信、高效廉洁政府

　　以法治思维规范政府行为，以改革创新促进政府职能转变，是新形势下加强政府自身建设的内在要求。各级政府要在党委的领导下，在法治轨道上开展工作，加快建设职能科学、权责法定、执法严明、公开公正、廉洁高效、守法诚信的法治政府。

　　深入推进依法行政。贯彻落实《中共中央关于全面推进依法治国若干重大问题的决定》，按照“法定职责必须为、法无授权不可为”的要求，推进政府机构、职能、权限、责任法定化，切实转变政府职能和管理方式。强化政府的程序规则，加强政府绩效管理，落实公众参与、专家论证、风险评估、合法性审查、集体讨论决定等重大行政决策法定程序。健全行政执法责任制、评议考核制和责任追究制，坚决惩处行政执法中不作为、乱作为和失职渎职行为。依法公开政务信息，推进行政决策公开、执行公开、管理公开、服务公开和结果公开。深入推进“六五”普法，抓好法治广东建设五年规划的落实。积极开展承接地方立法权相关准备工作。严格执行市人大及其常委会的决议和决定，加强与政协协商，自觉接受民主监督。

　　深化改革创新。坚持问题导向，深化行政审批制度改革，强化目录动态管理。推进行政执法体制改革，加强重点领域综合执法，减少市县政府执法队伍种类，着力解决权责交叉、多头执法问题。深化商事制度改革，推进“三证合一”，加快建设商事主体综合信息管理平台，强化后续监管。完善公共资源交易平台，加强重点领域监管。积极引进战略投资者参与国企改革。稳妥推进市、县、镇三级公务用车改革。加快机关事业单位养老保险制度和工资制度改革。推进梅州日报社、市广播电视台的集团化改革，争取年内完成经营服务类、公益三类事业单位分类改革任务。推进政府向社会组织购买公共服务。继续在政府工作部门开展“微改革、微创新”，建立健全市政府部门公开承诺制，提高执行力和办事效率。完善实体办事大厅、网上办事大厅和"12345"政府服务热线三大平台建设，继续开展“梅州发布”微访谈，拓宽政府和社会的沟通渠道。

　　坚持廉洁从政。严守政治纪律和政治规矩，严格落实中央八项规定和党风廉政建设责任制。深入开展“廉洁城乡”创建活动，推动全市廉洁文化建设。严格执行《预算法》，建立健全全面规范、公开透明的预算制度，继续压减“三公”经费。深化领导干部经济责任审计，加强对重点项目、重要资金和重大工程的行政监察和审计监督，坚决纠正各种不正之风。强化对重点工作和民生实事的督查督办，组织开展政风行风评议活动，坚决整治懒政、怠政行为。加强公务员教育培训和管理，建设一支勇担当、善作为、干净干事的公务员队伍，树立为民务实清廉的政府形象。

　　各位代表！今年，我们将继续推进十项惠民工程，让全市群众得到更多的实惠。一是提高底线民生保障水平。城乡居民基本养老保险基础养老金标准从每人每月80元提高到100元；城镇和农村低保补助人均月补差水平从334元、148元分别提高到375元和173元；五保对象供养标准提高到所在县（市、区）上年度农村居民人均纯收入的60%以上；孤儿集中供养水平从每月1150元提高到1240元，分散供养水平从每月700元提高到760元；残疾人生活津贴从每年600元提高到1200元，重残护理补贴从每年1200元提高到1800元。二是推进教育公平均衡发展。城乡免费义务教育生均公用经费补助标准，小学从950元提高到1150元，初中从1550元提高到1950元。中等职业学校和普通高中国家助学金标准，从每生每年1500元提高到2000元；市属民办、县属中等职业学校免学费补助标准，从每生每年2500元提高到3000元；市属公办中等职业学校免学费补助标准，从每生每年3000元提高到3500元。实施高中阶段残疾学生免学杂费、课本费政策。三是提高医疗卫生保障水平。基本公共卫生服务项目补助标准从每人每年35元提高到40元。城乡居民基本医疗保险补助标准从年人均320元提高到380元。基本医疗保险异地就医即时结算联网医院从6家增加到12家。建立市、县级疾病应急救助制度。四是提升养老服务水平。为80周岁以上老人、60周岁以上困难群体（低保、五保、优抚对象及“三无”人员）购买意外伤害保险。动工建设梅州市老年公寓（市养老示范中心）。实施城乡居民殡葬基本服务由政府免费提供政策，减免7项基本服务收费。五是优化生态环境。扩大省级生态公益林面积143万亩；规划建设18个森林公园、4个湿地公园；建设提升百岁山公园、马鞍山公园、泮坑公园和梅花山公园。六是加大保障性住房建设力度。新开工建设12364套（户）城市棚户区改造安置房（含货币安置补偿）和1523套（户）公共租赁住房（含租赁补贴），完成2570套（户）城市棚户区改造安置房建设。完成14034户农村危房和32户华侨农场危旧房改造。七是改善城乡居民生产生活条件。加大中心城区供水基础设施建设，动工建设城北自来水厂和梅城新水厂（首期10万吨）。开展巨灾保险改革试点工作。完成500公里新农村公路硬底化改造。建设17个中心镇污水处理厂。八是完善公共文化体育服务。建成60个农村文化俱乐部。实施公共体育场馆免费或低收费开放。九是提高城区交通便民水平。开通梅县区城市公交。城区新增100辆纯电动公交车，更新150辆清洁能源出租车。规划建设1-2个公共停车场。改造提升一批市政道路。开展交通秩序综合治理。十是加强公共法律服务。全面开展一村（社区）一法律顾问工作，对全市2242个村（社区）按每个不低于1万元的标准予以补助，以政府购买服务方式对村（社区）法律顾问公益性服务进行补贴。

　　各位代表！梅州已经踏上全面加快振兴发展的新征程，实现美好的愿景，需要我们勇于担当、真抓实干。让我们在省委、省政府和市委的坚强领导下，团结带领全市人民，调动一切积极因素，凝心聚力，务实苦干，确保全面完成全年各项目标任务！
